# AAV2 capsid clearance and neuronal trafficking dynamics in the central nervous system

**DOI:** 10.1038/s41434-026-00617-1

**Published:** 2026-04-30

**Authors:** Thanvi Gullapalli, Jake W. Willows, Ahmad Karkhah, Vikas Munjal, Abigail Nimmo, Kaya E. Ceyhan, Meika Travis, Allison O’Brien, Matthew T. Rocco, Anitvir S. Taunque, Kristy L. Townsend, Lluis Samaranch

**Affiliations:** 1 Undergraduate Research Program, The Ohio State University, Columbus, OH USA; 2 Department of Neurological Surgery, The Ohio State University, Columbus, OH USA; 3 Gene Therapy Institute, The Ohio State University, Columbus, OH USA; 4 Medical Student Research Program, The Ohio State College of Medicine, The Ohio State University, Columbus, OH USA; 5 Biomedical Sciences Graduate Program, The Ohio State University, Columbus, OH USA

**Keywords:** Cellular neuroscience, Targeted gene repair

## Abstract

Adeno-associated virus serotype 2 (AAV2) remains one of the most common vectors for CNS gene delivery. Yet, its long-term intraparenchymal trafficking and the relationship between capsid persistence and transgene expression remain poorly understood. In this study, we tracked the spatial and temporal patterns of AAV2 following direct striatal infusion in rats, analyzing tissue at 3 days, 3 weeks, 12 weeks, 30 weeks, and 67 weeks after injection. Using immunofluorescence for the human aromatic L-amino acid decarboxylase (AADC) transgene and an epitope detecting intact AAV2 capsids (A20), we mapped capsid localization, clearance, and axonal transport over more than a year. Following direct striatal infusion, AAV2 capsids rapidly localized to striatal neurons, with early accumulation in the substantia nigra pars reticulata (SNpr) detectable by 3 days post-infusion, but without evidence of nigral neuron transduction. Transgene expression increased over time and peaked locally at 12 weeks, with a delayed yet robust AADC signal in striatonigral terminals by 30 weeks. By 67 weeks, capsid signal was minimal while AADC expression remained stable. These findings clarify the long-term dynamics of AAV2 distribution, capsid persistence, and axonal transport in the adult brain, informing our understanding of vector behavior and durability following intraparenchymal AAV2 gene delivery.

## Introduction

Adeno-associated virus serotype 2 (AAV2) is one of the most extensively studied vectors for central nervous system (CNS) gene therapy and remains widely used in clinical applications that require stable neuronal transduction [1–12]. AAV2 is a small, non-enveloped parvovirus that packages approximately a 4.7 kb single-stranded DNA genome, with recombinant vectors retaining only the inverted terminal repeats needed for genome rescue, replication, packaging, and long-term episomal maintenance within host cells following transduction [13–15]. The mature capsid primarily interacts with heparan sulfate proteoglycans [16–18]. It utilizes additional coreceptors, such as fibroblast growth factor receptor 1 (FGFR1), integrins αVβ5 and α5β1, and the entry factor AAV receptor (AAVR), to enable internalization, endosomal trafficking, and nuclear delivery [19–23]. Following receptor binding at the cell surface, AAV2 uptake can occur through multiple endocytic pathways, including clathrin-mediated endocytosis and clathrin-independent routes such as the CLIC/GEEC pathway [24]. The relative utilization of these pathways depends on cell type and receptor engagement and initiates intracellular trafficking processes that strongly influence downstream transduction efficiency.

During endosomal maturation, the capsid undergoes pH-dependent conformational changes in the VP1/VP2 domains that facilitate endosomal escape. Escaped particles are then imported into the nucleus, where uncoating occurs [25–28]. Although the precise site and mechanism of uncoating remain unknown, in vitro studies show nuclear localization within approximately 2 h, while in vivo experiments have detected nuclear AAV2 capsids as early as 30 min after injection, depending on tissue and delivery conditions [29–33].

AAV vectors can also exploit neuronal connectivity through axonal transport, distributing the vector transgene beyond the injection site [34, 35]. Neurons can transport organelles and macromolecules between soma and distal terminals, supporting both anterograde and retrograde axonal transport of viral components [36, 37]. For AAV2, this process is mostly anterograde, consistent with previous studies showing transgene signal in the distal nerve terminals of transduced neurons [35, 38]. However, it remains unclear whether the transported material consists of intact capsids, partially uncoated particles, or soluble intermediates. Additionally, several studies have examined AAV2 distribution after intracranial infusion, but a detailed temporal map integrating capsid localization, capsid clearance, and transgene expression across short-term and long-term time points has not been systematically documented.

**Fig. 1:**
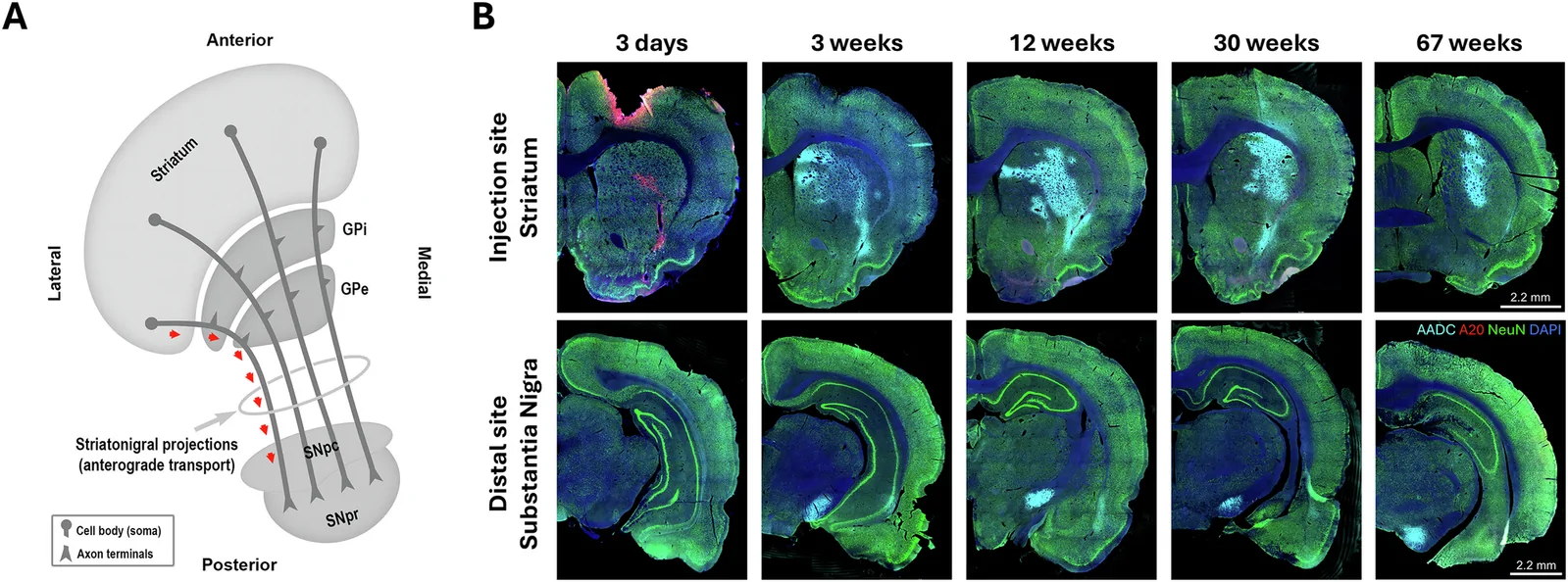
Striatonigral projections, and temporal evolution of AAV2 capsid distribution and hAADC expression in the rat striatum. Schematic of the anterograde projections between the striatum and the substantia nigra pars reticulata **A**. Representative immunofluorescence images of the rat striatum following convection-enhanced delivery of AAV2-hAADC, collected at 3 days, 3 weeks, 12 weeks, 30 weeks, and 67 weeks post-infusion **B**. Intact AAV2 capsids were detected using the A20 antibody, and transgene expression was assessed by immunolabeling for AADC. GPi, globus pallidus internus; *GPe*, globus pallidus externus, *SNpc*, Substantia nigra pars compacta; *SNpr*, Substantia nigra pars reticulata. Scale bars: 2.2 mm. *Note: Panel* ***A*** *is inspired by Grofova’s 1979 schematics (ISBN 978-0-08-023174-7)*.

In this study, we performed a comprehensive longitudinal analysis of AAV2-hAADC following convection-enhanced delivery into the rat striatonigral pathway (Fig. 1A). Using immunolabeling for AADC and the A20 epitope on intact AAV2 capsids, we assessed vector distribution at 3 days, 3 weeks, 12 weeks, 30 weeks, and 67 weeks post-injection. Our findings reveal a clear spatiotemporal dissociation between the persistence of intact AAV2 capsids and the kinetics of transgene expression. We observed robust early intact capsid detection followed by a progressive decline, contrasted by a sustained rise in AADC expression both locally within the striatum and distally within its striatonigral projection terminals. Notably, although intact AAV2 capsids reached these distal terminals, we found no evidence of transneuronal transduction in the substantia nigra pars reticulata, strongly supporting the view that AAV2-based transgene anterograde transport is non-transsynaptic.

## Results and discussion

**Fig. 2:**
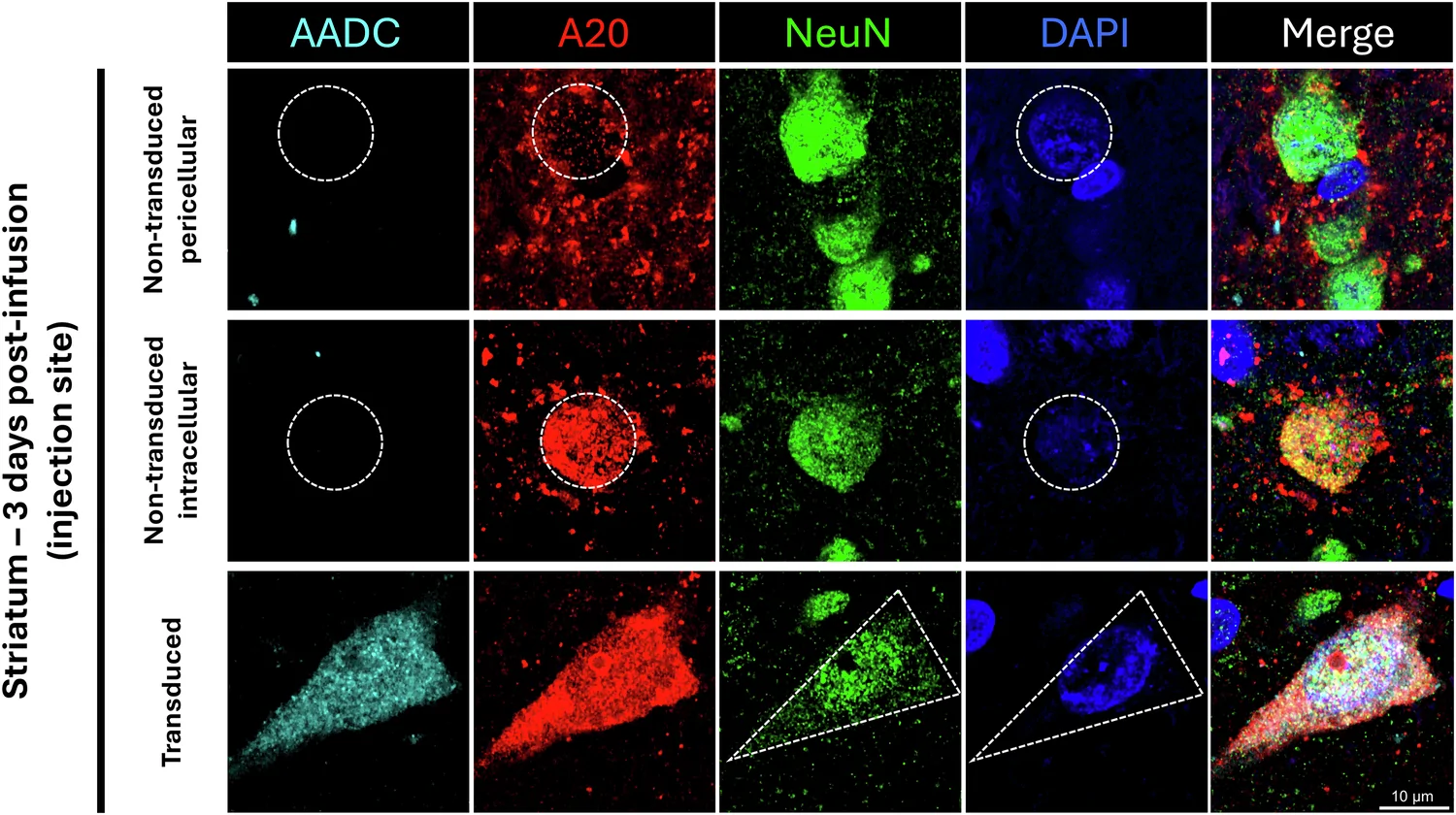
Early pericellular and intracellular localization of AAV2 capsids at 3 days post-infusion. High-magnification confocal images of the striatum 3 days after AAV2-hAADC infusion showing detailed localization of AAV2 capsids. A20 immunoreactivity was observed predominantly in pericellular and extracellular patterns, with detectable intracellular signal in a subset of neurons, indicating early stages of capsid internalization. AADC expression at this time point was sparse, consistent with limited productive transduction. Scale bar: 10 μm.

Three days after infusion, immunohistochemical detection with the A20 antibody demonstrated widespread distribution of intact AAV2 capsids throughout the striatal parenchyma, whereas expression of the transgene product, the AADC enzyme, remained minimal (Fig. 1B). Higher magnification revealed that capsids localized predominantly around neuronal somata, although occasional intracellular signal was observed. Because A20 recognizes both surface-bound and internalized intact capsids, these findings indicate that viral uptake begins as early as 3 days post-infusion (Fig. 2). Limited transgene expression was detectable, although it was rare at this early time point, indicating that productive transduction had not yet occurred in most cells. This spatial distribution supports the conclusion that neuronal uptake of AAV2 is already underway, even though detectable protein levels remain low. The delay between capsid internalization and transgene expression is consistent with previous studies showing that AAV2 must undergo endosomal escape, cytoplasmic trafficking, nuclear entry, and second-strand synthesis before gene expression can commence [28, 35, 39]. The strong A20 signal, not overlapping with NeuN or DAPI signal, suggests extracellular localization, likely reflecting binding of AAV2 to heparan sulfate proteoglycans (HSPGs) and other extracellular matrix components that mediate initial attachment [16]. These glycosaminoglycans, together with laminin and fibronectin, can transiently bind and hold viral capsids within the interstitial space. Such reversible, non-fusogenic interactions help stabilize capsids in the interstitial space prior to engagement with coreceptors that promote endocytosis [16].

**Fig. 3:**
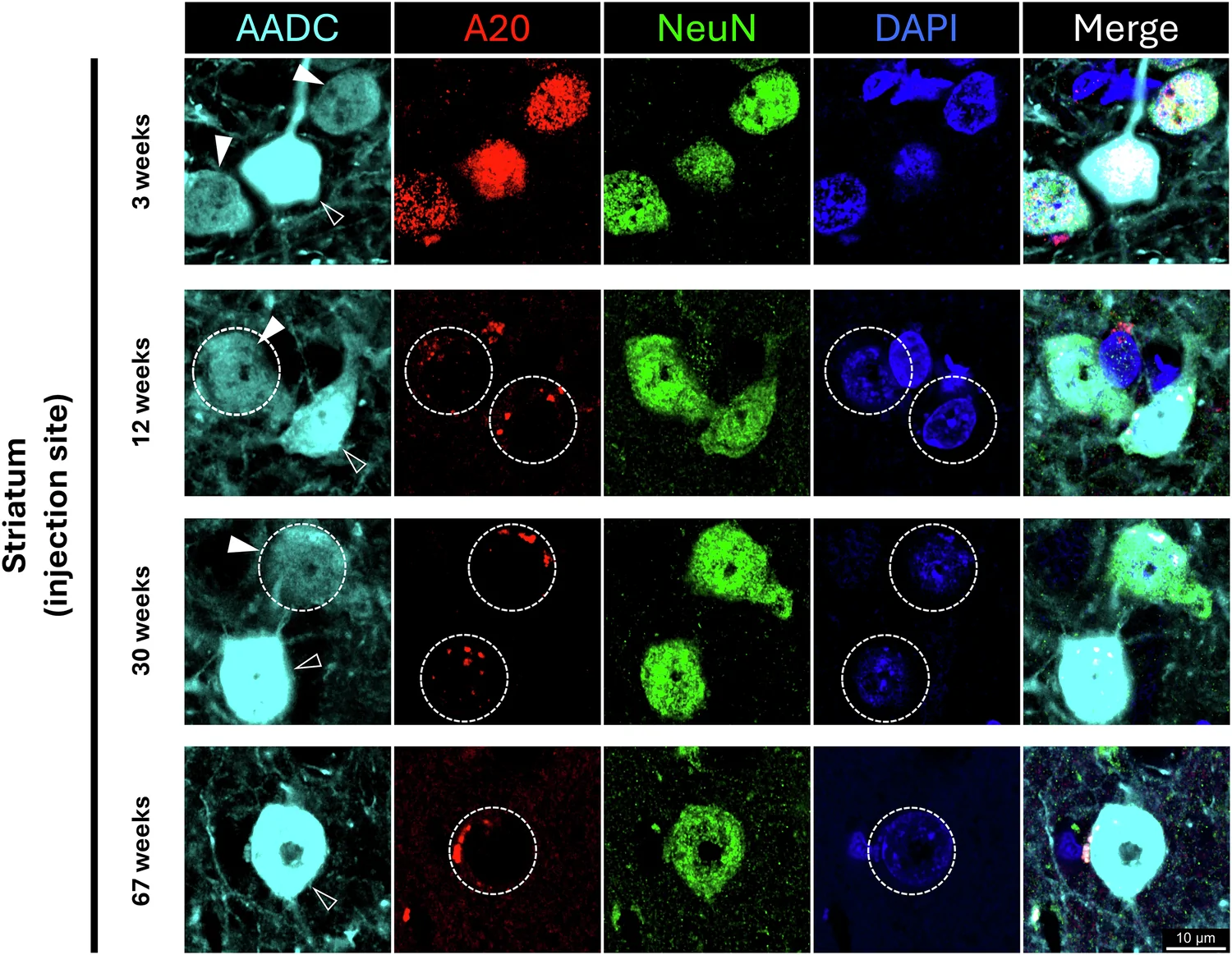
Long-term persistence of nuclear AAV2 capsids and sustained hAADC expression in the striatum. Representative striatal sections at 3 weeks, 12 weeks, 30 weeks, and 67 weeks post-infusion illustrating the progressive restriction of A20 signal to intracellular, predominantly nuclear compartments, alongside robust and sustained AADC expression. Extracellular A20 immunoreactivity was minimal at later time points. Scale bar: 10 μm.

By 3 weeks post-infusion, A20 immunoreactivity in the striatum had markedly decreased, coinciding with a robust increase in AADC enzyme expression (Fig. 1B). This temporal shift indicates efficient capsid uptake, intracellular trafficking, uncoating, and transcriptional activation of the vector genome. AAV2 is known for high endocytic efficiency, with up to 80% of virions internalized through multiple endocytic pathways, including clathrin-mediated and clathrin-independent mechanisms such as the CLIC/GEEC pathway [24]. At higher magnification, A20 signal was primarily overlapping with DAPI and NeuN-positive regions, with minimal extracellular signal remaining (Fig. 3). No non-neuronal transduction was observed, consistent with previous reports demonstrating preferential neuronal targeting of AAV2 in the brain [40]. Following internalization, AAV2 capsids must traffic through the endosomal system, escape into the cytosol, translocate to the nucleus, and uncoat before transcription can begin [26, 41]. Then, AAV2 particles traffic through the endosomal system and must undergo endosomal escape prior to nuclear entry. Emerging evidence suggests that AAV2 may engage the AAVR and traffic through retrograde pathways involving the trans-Golgi network before reaching the nucleus, suggesting a more complex intracellular routing process than previously appreciated [42]. The relative contribution of these pathways may depend on cell type and experimental context and remains an active area of investigation.

The reciprocal relationship between declining A20 signal and rising AADC enzyme expression during the 3-day to 3-week period highlights a critical processing window in which AAV2 particles transition from extracellular capsids to productive transduction.

At twelve weeks post-infusion, strong and widespread AADC enzyme expression persisted throughout the striatal parenchyma (Fig. 1B). A20 immunoreactivity was further reduced overall and largely confined to low-intensity nuclear staining (Fig. 3). These findings suggest that most AAV2 capsids underwent successful internalization and uncoating, with only residual intranuclear capsids remaining. The sustained AADC signal aligns with prior studies showing that AAV2 genomes persist in non-dividing neurons as episomal structures capable of long-term transgene expression [43–45]. Whether the continued decline in A20 signal reflects exclusively productive processing or alternative degradation pathways remains unclear, as both proteasomal and lysosomal mechanisms have been implicated in AAV2 capsid turnover [46]. The role of the ubiquitin-proteasome system in modulating AAV2 transduction has been well documented, with proteasome inhibitors shown to enhance nuclear trafficking and transgene expression [47]. This modulation may reflect the inherently low immunogenicity of AAV2 within immune-privileged sites like the brain, as accelerated capsid degradation and antigen presentation on MHC class I molecules are diminished, thereby effectively mitigating robust cytotoxic T cell responses [48]. Future experiments using co-labeling with endosomal, lysosomal, and proteasomal markers will be necessary to determine whether the residual capsids represent stalled intermediates or slow-degradation products.

At thirty weeks post-infusion, AADC enzyme expression remained strong and comparable to the 12-week group indicating stable transduction and continued episomal vector activity (Fig. 1B). Although extracellular A20 signal was nearly absent, A20 signal colocalizing with DAPI persisted within neurons (Fig. 3). The long-term presence of intact capsids likely reflects the inherent structural stability of the AAV2 capsid, which is highly resistant to degradation in postmitotic cells [49]. Persistent intranuclear capsids may represent slow-uncoating particles, incomplete processing intermediates, or capsids sequestered in nuclear compartments with limited access to degradative pathways. Because nuclear-localized capsids are less likely to enter cytoplasmic proteasomal processing pathways, they may be less available for MHC-I antigen presentation, although the immunological significance of this phenomenon in the CNS remains unresolved. Additional studies will be required to determine whether retained capsids influence long-term vector stability or immune visibility.

In the long-term follow-up animal (*n* = 1) analyzed at 67 weeks post-infusion, both A20 and AADC staining patterns were comparable to those observed in the 12- and 30-week groups (Fig. 3). Although limited by sample size, this observation suggests that AAV2-mediated transduction and the persistence of residual capsids can remain stable for more than a year following parenchymal delivery to the CNS.

**Fig. 4:**
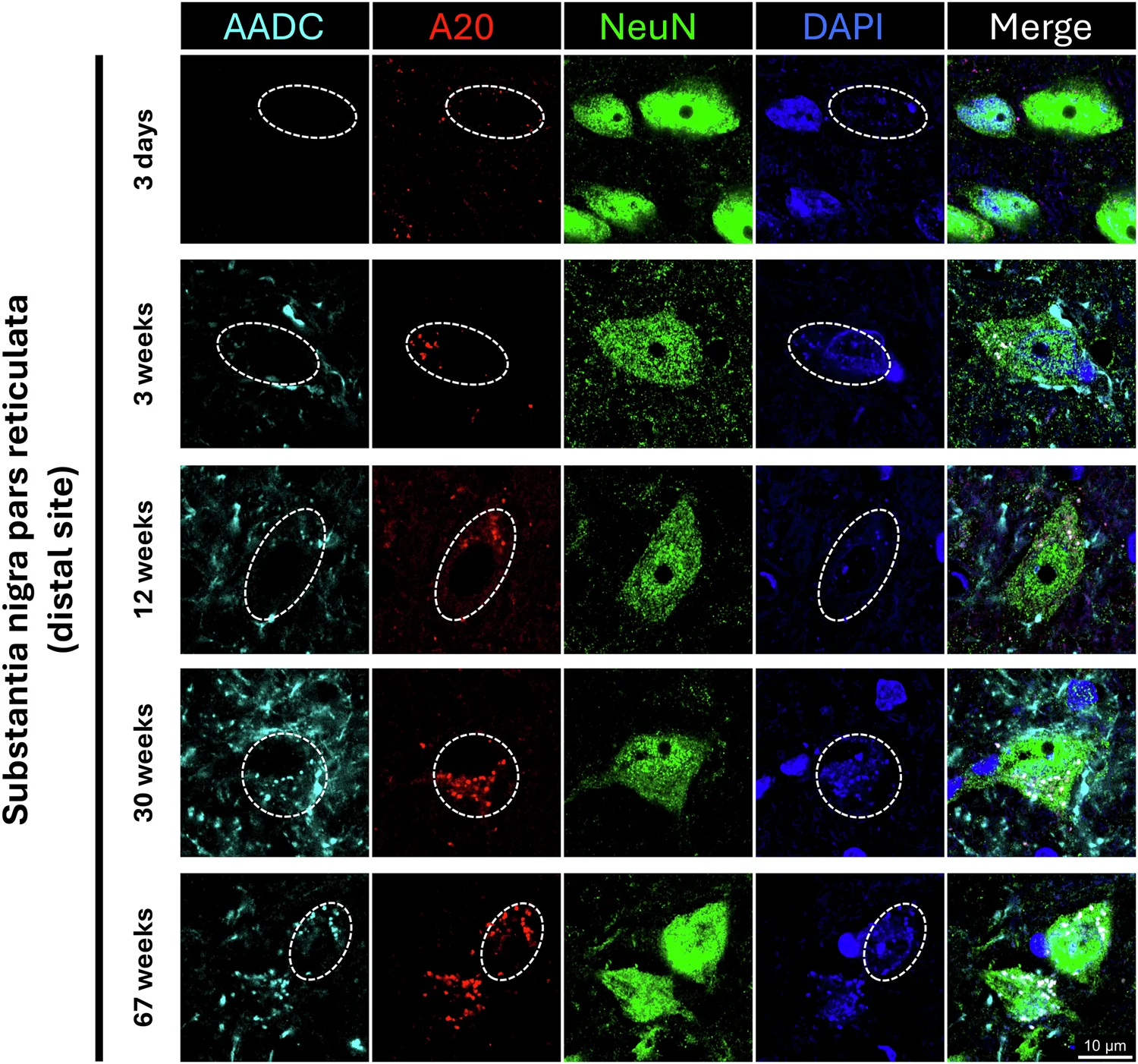
Anterograde axonal transport of AAV2 capsids to the substantia nigra pars reticulata without trans-synaptic transduction. Immunofluorescence analysis of the substantia nigra pars reticulata (SNpr) at 3 days, 3 weeks, 12 weeks, 30 weeks, and 67 weeks following intrastriatal AAV2-hAADC infusion. A20-positive capsids were detected in the SNpr with a delayed but progressive increase over time, consistent with anterograde axonal transport from striatal projection neurons. AADC expression was detectable in the striatal terminals at SNpr levels at later time points. However, no AADC-positive neuronal cell bodies were observed, indicating absence of trans-synaptic transduction. Scale bar: 10 μm.

To determine whether similar temporal patterns occurred in anatomically connected regions, we next examined the substantia nigra pars reticulata (SNr), the primary downstream target of striatal GABAergic projections (Fig. 1A). As expected, no AADC-positive signal was detected in the SNr at 3 days post-infusion. However, AADC expression gradually increased at later time points, becoming detectable by 3 weeks and remaining stable through 30 and 67 weeks (Fig. 4). This delayed appearance likely reflects trafficking of newly synthesized AADC protein from striatal neurons to their distal axon terminals.

In contrast, A20 immunoreactivity in the SNr followed a distinct temporal profile, with minimal presence at 3 days but progressively increasing at 12, 30, and 67 weeks. This pattern diverges from the declining A20 signal in the striatum and is consistent with progressive accumulation of intact capsids at striatal axon terminals. These findings support prior evidence for anterograde axonal transport within the striatonigral pathway [35, 38]. Once at presynaptic terminals, capsids may be retained within vesicular compartments, dock at presynaptic membranes, or undergo limited exocytic release. Despite the presence of capsids in the SNr, no AADC-positive SNr neurons were observed at any time point (Fig. 4), confirming that AAV2 does not efficiently undergo transneuronal transport at any assessed stage. This aligns with prior evidence showing that AAV2 lacks the molecular determinants required for anterograde trans-synaptic mobility, unlike serotypes such as AAV1 that can undergo synaptic crossing [50]. Specifically, AAV2 capsids are not fusogenic and do not engage with the vesicular release pathways exploited by naturally trans-synaptic viruses, precluding their exit from presynaptic terminals. Additionally, the strong HSPG-binding profile of AAV2 promotes sequestration at axon terminals and may restrict extracellular mobility, further limiting access to traverse synaptic clefts.

## Conclusions

This study provides a comprehensive temporal map of AAV2 capsid distribution, clearance, and transgene expression following direct parenchymal delivery to the rat striatum. By integrating A20 immunoreactivity with AADC expression across five time points, we delineate a sequential progression of viral entry, intracellular trafficking, axonal transport, and sustained transgene expression that has not previously been characterized at this level of resolution.

Our findings demonstrate that AAV2 remains predominantly localized to the injection site during the early phase, with capsid signals declining as AADC expression increases, consistent with productive uncoating and transcriptional onset. The delayed yet robust accumulation of AADC-positive axons in the substantia nigra, coupled with the presence of A20-positive puncta but absence of AADC-positive nigral somata, strengthens the evidence that AAV2 undergoes long-distance axonal transport without trans-synaptic transduction of second-order neurons. This dissociation between transported capsid and localized transcription resolves one of the long-standing uncertainties surrounding AAV2 trafficking in mature CNS circuits. The extended timepoints also reveal that AAV2-mediated transgene expression remains stable for well over a year, with no evidence of vector spread beyond expected projection pathways.

Overall, this work advances the understanding of AAV2 biology in the adult brain by defining the kinetics sequence of viral trafficking and transgene expression over long timescales. These insights may support the rational design, optimization, and interpretation of AAV2-based gene therapy programs targeting the human CNS.

## Materials and methods

### Viral vector and experimental design

Thirteen female Sprague-Dawley rats ( ~ 200-300 g) underwent bilateral striatal injections of an AAV2 vector encoding the cDNA of the human aromatic L-amino acid decarboxylase gene (AAV2-hAADC), under the control of the cytomegalovirus promoter, using convection-enhanced delivery. The animals were randomly assigned to different survival times: three days (*N* = 3), three weeks (*N* = 3), 12 weeks (*N* = 3), 30 weeks (*N* = 3), and 67 weeks (*N* = 1).

The viral vector was produced at The Children’s Hospital of Philadelphia, with a total titer of 8.28E + 12 vg/mL. All procedures followed The Ohio State University Institutional Animal Care and Use Committee guidelines.

### Surgical procedure and vector infusion

Before vector infusion, the animals were housed on a 12:12 h light/dark cycle, with room temperature and humidity maintained at 19–21 °C and 50–60%, respectively. All animals had ad libitum access to food and water. The animals were anesthetized with 1–3% isoflurane and received subcutaneous injections of 0.1 mL of 1:10 diluted Buprenorphine and Meloxicam. The skull was exposed, and two burr holes were made at the level of the striatum. A total of 5 μL of AAV2-hAADC was infused per hemisphere using a custom-made reflux-resistant cannula (O.D. 235 mm; I.D. 100 mm - Polymicro Technologies) at a rate of 1 μL/min for 5 min, based on predefined stereotactic coordinates: anteroposterior (A/P) + 0.5 mm,mediolateral (M/L) + 3.0 mm, ventrodorsal (V/D) -5.0 mm relative to bregma and dura. Following vector infusion, the cannula was left in place for 2 min to minimize reflux along the needle tract. The scalp incision was then sutured, and animals were allowed to recover before being returned to their housing and monitored during the postoperative period.

### Tissue processing

At the designated survival times, animals were deeply anesthetized and transcardially perfused with heparinized phosphate-buffered saline (hPBS), followed by ice-cold 4% paraformaldehyde in PBS (PFA/PBS). The brains were harvested and post-fixed in 4% PFA/PBS for 24 h at 4 °C, followed by immersion in 30% sucrose. The blocks were frozen and sectioned into 40 μm-thick coronal sections using a sliding microtome. These sections were collected in cryoprotectant and stored at −20 °C until further processing. Selected sections were processed for immunofluorescence staining using a free-floating technique.

### Immunofluorescence staining

To assess the dynamics and distribution of free capsids and AADC-transduced cells in the injection site and distal regions, triple immunofluorescence staining for hAADC (transgene), A20 (intact capsid marker), and NeuN (neuronal marker) was conducted. Briefly, sections were washed three times in PBS/0.1% Tween-20 for 5 min each and then incubated in a blocking solution (1x Animal Free Blocker, Vectro Lab SP-5030) for 1 h at room temperature. Next, sections were incubated overnight at 4 °C with primary antibodies: AADC antibody (1:500; rabbit AB136, Millipore), A20 antibody (1:500; mouse 03-65155, American Research Products), and NeuN antibody (1:500; chicken MAB377, Millipore). The following day, sections were washed in PBS/1% Tween solution and incubated with secondary antibodies for 1 h at room temperature (Anti-rabbit, 1:500; Alexa Fluor-555 ref: A32794, Invitrogen. Anti-mouse, 1:500; Alexa Fluor-488 ref: A32766, Invitrogen. Anti-chicken, 1:500; Alexa Fluor-647 ref: A78952, Invitrogen). Finally, sections were mounted on slides, coverslipped with DAPI-Fluoromount G (ref: 0100-20, Southern Biotech), and evaluated.

It should be noted that the A20 antibody detects assembled capsids independent of genome content. Therefore, A20 immunoreactivity may reflect both genome-containing and empty particles. Although affinity chromatography-based purification methods used during vector production are designed to enrich for genome-containing particles, they could contribute to early capsid signal or modulate local immune responses.

### Confocal microscopy

All photomicrographs were captured using a Leica Stellaris 5 confocal microscope. Excitation and emission spectra were tuned for each fluorophore, and multiple channels were scanned sequentially. Fluorescent labels were excited with either a 405 nm diode laser or a white-light laser. The field of view was scanned bidirectionally at 700 Hz with Pinhole Airy set at 1.00 AU. Photons were detected with Power HyD S detectors, and scanned lines were averaged five times. Mosaics of brain sections were tiled maximum-intensity projected z-stacks (3 μm step size, 1024×1024 pixel ratio per tile) captured using a 10X objective (HC PL APO 10×/0.40 CS2) with a 1.28X confocal zoom applied. After visually scanning entire brain regions of interest, representative images of individual cells (5 images per region, per timepoint) were captured at 63X objective magnification (HC PL APO 63×/1.40 OIL CS2) at 2048×2048 pixel aspect ratio. A 1.28X confocal zoom and a further 4.06X digital zoom were applied to improve visualization of individual cell bodies. Image processing was performed using Leica Application Suite X and Fiji software. Subcellular localization was inferred based on spatial overlap of A20 immunoreactivity with DAPI (nuclear marker) and NeuN (neuronal marker) in confocal optical sections.

## Data Availability

Data and/or specimens are available from the corresponding author upon reasonable request and in accordance with The Ohio State University requirements and approval.
